# Adenocarcinoma risk in gastric atrophy and intestinal metaplasia: a systematic review

**DOI:** 10.1186/s12876-017-0708-4

**Published:** 2017-12-11

**Authors:** Andrew D. Spence, Chris R. Cardwell, Úna C. McMenamin, Blanaid M. Hicks, Brian T. Johnston, Liam J. Murray, Helen G. Coleman

**Affiliations:** 1 Cancer Epidemiology and Health Services Research Group, Centre for Public Health, Queen’s University Belfast, Belfast, Northern Ireland, UK; 2 Belfast Health and Social Care Trust, Belfast, Northern Ireland, UK

**Keywords:** Gastric cancer, Gastric atrophy, Intestinal metaplasia, Progression, Premalignant

## Abstract

**Background:**

Gastric cancer (GC) has a poor prognosis with wide variation in survival rates across the world. Several studies have shown premalignant lesions gastric atrophy (GA) and intestinal metaplasia (IM) influence gastric cancer risk. This systematic review examines all available evidence of the risk of GC in patients with GA or IM and explores the geographical variation between countries.

**Methods:**

EMBASE, MEDLINE, Web of Science and the Cochrane Library were searched for relevant articles published to June 2016 investigating the risk of GC in individuals with GA or IM. Analysis was performed to determine variation based on geographical location. Study quality was assessed using the Newcastle-Ottawa Scale and heterogeneity between studies was also evaluated.

**Results:**

Fifteen relevant articles were identified, in which there were eight studies of GC incidence in GA and nine in IM cohorts (two articles investigated both GA and IM). The incidence rate of GC in patients with GA ranged from 0.53 to 15.24 per 1000 person years, whereas there was more variation in GC incidence in patients with IM (0.38 to 17.08 per 1000 person years). The greatest GC incidence rates were in Asian countries, for patients with GA, and the USA for those with IM (15.24 and 17.08 per 1000 person years, respectively). The largest studies (four over 25,000 person years) had an incidence rate range of 1.0–2.5 per 1000 person years, however, in general, study quality was poor and there was marked heterogeneity.

**Conclusion:**

Overall there is a wide variation in annual incidence rate of GC from premalignant lesions. With the recent introduction of surveillance guidelines for gastric atrophy and intestinal metaplasia in the Western world, future assessment of this risk should be performed. Furthermore, substantial heterogeneity supports the need for more robust studies in order to pool results and determine the overall incidence rate of gastric cancer for patients with these premalignant lesions.

**Electronic supplementary material:**

The online version of this article (10.1186/s12876-017-0708-4) contains supplementary material, which is available to authorized users.

## Background

Gastric cancer (GC) is the fifth most common malignancy and third leading cause of death from cancer worldwide [1]. There is geographical variation in GC incidence, with Asia being the most common region, with lower rates in USA and Europe where, although incidence has been decreasing in recent decades, the 5-year survival remains poor (24% in Europe) [2]. In Japan and Korea, high incidence and historically poor survival rates for gastric cancer have led to the introduction of surveillance programmes, which have increased 5-year survival to 60%, prompting suggestions to introduce such programmes in Western countries [3]. However, the lower incidence of Helicobacter pylori (*H. pylori*), the most common contributing factor to GC, [4] in these lower risk areas, has impacted surveillance programme feasibility. In addition to *H. pylori*, chronic inflammation of the stomach mucosa results in atrophic changes, including loss of structured glandular cells, which are replaced by intestinal-type epithelium, pyloric-type glands and fibrous tissue [5]. The resultant gastric atrophy (GA) and intestinal metaplasia (IM) are known premalignant lesions for stomach cancer [6, 7].

Studies have shown a significantly increased risk of GC in patients with either GA (5.8 times the risk of GC compared to patients without GA) [8] or IM (10 times risk of GC compared to those individuals with no evidence of IM) [9]. Several other studies have reported the association of these lesions with GC; however, the design and quality of these studies are varied, [10–12] resulting in a wide range of observed cancer risk estimates.

To date, one systematic review has been published on the risk of GC in patients with GA, which used a serological method for diagnosis of these lesions [13]. The aim of our systematic review was to determine risk of progression to GC in patients with histologically confirmed GA or IM and assess the quality of published studies.

## Methods

### Search strategy

This systematic review was conducted in line with PRISMA guidelines [14] and the protocol was registered with PROSPERO (PROSPERO 2015:CRD42015022037) [15]. Ovid MEDLINE (US National Library of Medicine, Bethesda, Maryland), EMBASE (Reed Elsevier PLC, Amsterdam, the Netherlands), Web of Science (Thomson Reuters, USA), and The Cochrane Library were searched for relevant studies from inception to 10th June 2016. The search included publications in any language, was limited to humans and excluded reviews. The Keyword search terms and Medical Search Headings used in the MEDLINE search are described in Additional file 1: Table S1. Similar searches were conducted in other databases.

### Study selection

Independent screening of the titles and abstracts was conducted by the corresponding/first author (ADS) and one co-author (CRC, ÚCMcM, BMH, LJM or HGC) to determine eligibility for inclusion. A further search was conducted by hand searching reference lists of included studies. At each stage of the review process, discrepancies were resolved by discussion with a third author.

Studies were eligible for inclusion if they were observational cohort or interventional study designs. The study patients must have been adults who had a histological diagnosis (solely serological methods of diagnosis were not considered, as this technique is not widely used in Western countries) of GA or IM at endoscopy. Studies were required to have a minimum of 100 cases of GA or IM, and to report the incidence rate of GC and 95% confidence intervals (CI), or provide enough information to allow these to be calculated. In the original protocol, studies which included GC cases within 6 months of GA/IM were to be excluded from the review (as these could be prevalent cases), however this was used as a criteria in the study quality assessment rather than exclusion criteria. In the instance where two studies may be included but used the same sample population we will select the study with the longest follow-up/the one which allows us to calculate the incidence rate of GC.

### Data extraction

A data extraction form was developed following piloting on five included publications. Data were extracted from all included studies and statistical risk estimates were verified. Where available, data were extracted from included studies on characteristics of study participants (location of study, demographic and lifestyle factors of study population, timing of recruitment), follow-up period (including person-years, if provided), number of GC and GA or IM cases, proportion of participants who were H. pylori positive, identification and verification methods to determine GC and GA/IM cases.

### Quality assessment

Quality of individual studies was assessed using a modified version of the Newcastle-Ottawa Scale [16]. Two sections of this scale did not apply to this systematic review topic (selection of the non-exposed cohort and the comparability section), therefore studies could achieve a maximum score of six (rather than nine) points. Two independent assessors (ADS and HGC) scored all studies, with discrepancies resolved by discussion.

### Statistical analysis

The primary summary measure to describe risk of GC was incidence rate per year in GA or IM populations. Where the risk of progression of GA or IM to GC was not reported, person-years were calculated using the number of participants multiplied by mean follow-up in years. In five studies the mean was not reported and therefore the median follow-up was used instead ([11, 12, 17–19]. Poisson distribution was used to determine 95% CI for the rate of progression from premalignant lesion to GC.

Review Manager 5.3 (The Nordic Cochrane Centre, The Cochrane Collaboration, Copenhagen, Denmark) was used to present, graphically, the incidence rates in each study. It was planned overall meta-analysis for pooled incidence rate would be performed, and sensitivity analyses conducted, as per our protocol. This included subgroup analyses by geographical study location, age and sex of participants. Additional subgroup analyses, including GA/IM location, tumour site and histological subtype were also planned but too few studies reported on these to facilitate analyses. However, due to high clinical heterogeneity between studies (including high statistical heterogeneity, estimated using the *I* ^2^ statistic), [20] the results of meta-analyses are not presented. To determine if particular studies contributed significantly to the high heterogeneity sensitivity analyses were conducted excluding individual studies in turn. However, similar to the overall analysis, heterogeneity remained high, precluding meta-analyses. Publication bias was evaluated using a funnel plot, showing the standard error of log incidence against each study’s incidence rate.

## Results

### Summary

**Fig. 1:**
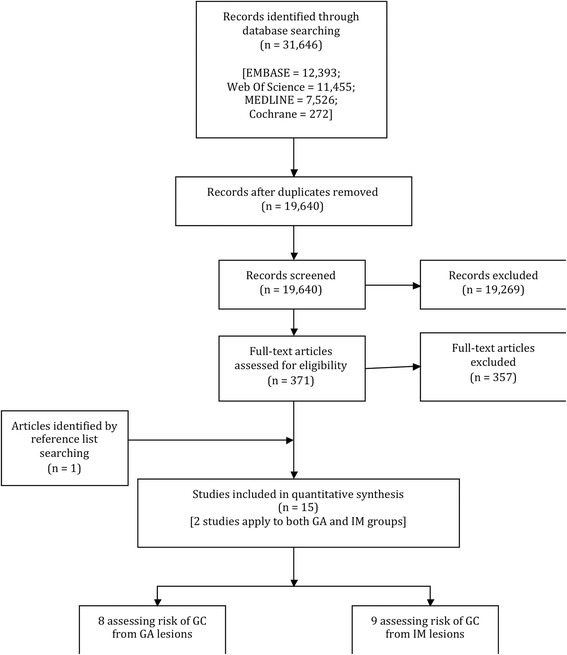
Flow chart demonstrating the search strategy and selection of included studies

As shown in Fig. 1, the search strategy resulted in 19,640 unique articles, of which 371 were selected for full text review. Following two stages of screening of full-text articles for eligibility, 15 publications were selected for inclusion, of which eight reported on GA and nine reported on IM patient groups (two publications contained studies of both GA and IM with GC risk). Four of the 15 publications included in this review were based in the United States of America, three in Japan, one in Korea and the remainder within European countries (Tables 1 & 2).

**Table 1:** Characteristics of studies included in the systematic review of gastric cancer incidence in patients with gastric atrophy

First author (Year) [reference]	Country	Study design	Indication(s) for endoscopy	Study follow up period	Outcome source	Number of patients in cohort	Male (%)	Mean age (years)	Follow-up (years) [mean/median]	HP* positive (%)
González (2016) [38]	Spain	Retrospective cohort	NR*	1995–2013	Pathology records, clinical records, endoscopy	156	46	49	12 (mean)	80
Inoue (2000) [10]	Japan	Prospective cohort	NR*	1985–1999	Hospital records, Cancer registry, mail survey	4397	48	50	10 (mean)	NR*
Lahner (2015) [11]	Italy	Prospective cohort	Surveillance programme	1992–2009	Endoscopy	200	33	55	7.5 (median)	NR*
Siurala (1974) [35]	Finland	Prospective cohort	NR*	1950–1973	Endoscopy	116	NR*	64	21 (mean)	NR*
Song (2015) [36]	Sweden	Retrospective cohort	Dyspepsia	1979–2011	Endoscopy, Cancer registry, ONS*	14,285	55	60	10.1 (mean)	NR*
Takata (2007) [25]	Japan	Prospective cohort	NR*	1991–2001	NR*	101	58	56	5.2 (mean)	NR*
Tatsuta (1993) [8]	Japan	Retrospective cohort	NR*	1968–1976	Cancer registry, pathology database	194	80	NR*	19 (mean)	100
Vannella (2010) [17]	Italy	Prospective cohort	NR*	1992–2008	Endoscopy	300	32	54	4.3 (median)	6

*NR Information not available, *HP* Helicobacter pylori, *ONS* Office for National Statistics

**Table 2:** Characteristics of studies included in the systematic review of gastric cancer incidence in patients with intestinal metaplasia

First Author (year) [reference]	Country	Study Design	Indication for Endoscopy	Study follow up period	Outcome Source	Number of patients in cohort	Male (%)	Mean age (years)	Follow-up (years) [mean/median]	HP* positive (%)
Bleibel (2003) [39]	United States of America	Retrospective cohort	NR*	1993–2012	Cancer registry	675	49	61	5.3 (mean)	18
De Vries (2010) [40]	The Netherlands	Prospective cohort	NR*	2006–2007	Endoscopy	101	50	61	2.3 (mean)	18
González (2016) [38]	Spain	Retrospective cohort	NR*	1995–2013	Pathology records, clinical records, endoscopy	467	56	53	12 (mean)	56
Horsley-Silva (2015) [26]	United States of America	Retrospective cohort	Dyspepsia	2003–2004	NR*	200	50	68	4.1 (mean)	19
Kim (2008) [27]	Korea	Prospective cohort	NR*	1992–1998	Endoscopy	515	88	45	10.2 (mean)	78
Li (2016) [18]	United States of America	Retrospective cohort	NR*	1997–2013	Cancer registry, clinical records	4146	48	66	7.1 (median)	51
Reddy (2014) [19]	United States of America	Retrospective cohort	NR*	2000–2011	Cancer registry	907	NR*	68	4.6 (median)	NR*
Song (2015) [36]	Sweden	Retrospective cohort	NR*	1979–2011	Endoscopy, Cancer registry, ONS*	11,530	55	66	7.9 (mean)	NR*
Tava (2006) [12]	Italy	Retrospective cohort	Dyspepsia	1989–1997	Endoscopy	259	51	60	3.9 (median)	27

*NR Information not available, *HP* Helicobacter pylori, *ONS* Office for National Statistics

### Incidence of gastric cancer in gastric atrophy patients

**Fig. 2:**
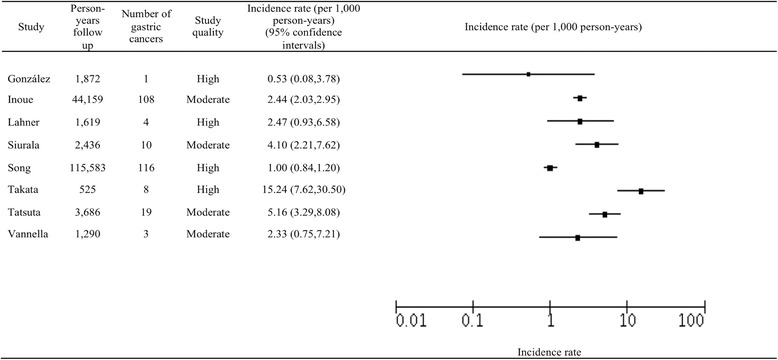
Analysis of studies of gastric cancer incidence in patients with gastric atrophy

The total number of patients in the GA cohorts was 19,749, in who 261 progressed to GC over a total of 171,170 person-years of follow-up (mean 21,396). The range of GC incidence in patients with GA was from 0.53 to 15.24 per 1000 person-years, as shown in Fig. 2. The majority of studies had an incidence rate of 1.0 to 5.2 per 1000 person-years (six of the eight studies), with the remaining two studies showing more extreme results. In further analyses, there was significant heterogeneity between studies (*I* ^2^ statistic of 94%), which precluded calculation of a pooled estimate.

### Incidence of gastric cancer in intestinal metaplasia patients

**Fig. 3:**
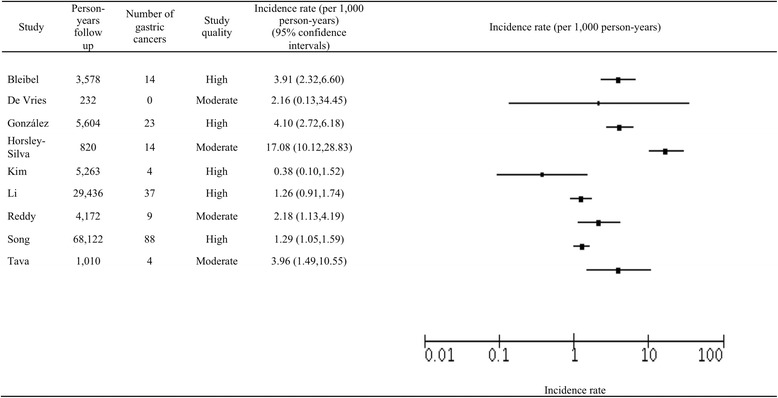
Analysis of studies of gastric cancer incidence in patients with intestinal metaplasia

There were a total of 18,800 patients in the IM cohorts, in who 193 developed GC over a total of 118,237 person-years of follow-up (mean 13,137). The range of GC incidence in patients with IM was from 0.38 to 17.08 per 1000 person-years (Fig. 3). Similar to the distribution of incidence rates for the studies of patients with GA there were two more extreme results, whereas seven of the nine studies had incidence rates between 1.2 and 4.1 per 1000 person-years. Horsley-Silva’s study, where there was a substantially higher incidence rate, had a follow up of 820 person-years, contrasting with the average follow up for the remaining studies of 16,774 person-years. Again, there was a substantial degree of heterogeneity (*I* ^2^ statistic of 93%) between studies of IM patients, therefore a pooled estimate was not calculated.

### Subgroup and sensitivity analysis

Table 3 shows the results from subgroup analysis. In terms of location, the largest estimate for GA was 4.10 per 1000 person-years in European studies, compared with 15.24 per 1000 person-years in those based in Asia. The largest estimate for IM studies based in Europe was 17.08 per 1000 person-years, comparable to the largest incidence rate for patients with GA in Asian countries. Patients with IM in Asia had a low incidence rate for GC development (0.38 per 1000 person-years), however there was only one study in this region.

**Table 3:** Subgroup analysis for studies of gastric cancer incidence in patients with gastric atrophy or intestinal metaplasia

Variable		Gastric atrophy		Intestinal metaplasia	
		Number of studies (references)	*I* ^2^ (%)	Number of studies (references)	*I* ^2^ (%)
Age (mean, years)	≥55	4^(^[11, 25, 35, 36]^)^	96	7^(^[12, 18, 19, 26, 36, 39, 40]^)^	94
	<55	3^(^[10, 17, 38]^)^	13	2^(^[27, 38]^)^	90
Male (% study population)	≥50	3^(^[8, 25, 36]^)^	98	6^(^[12, 26, 27, 36, 38, 40]^)^	95
	<50	4^(^[10, 11, 17, 38]^)^	0	2^(^[18, 39]^)^	92
Country/Region	Asia	3^(^[8, 10, 25]^)^	94	1^(^[27]^)^	NA*
	Europe	5^(^[11, 17, 35, 36, 38]^)^	82	4^(^[12, 36, 38, 40]^)^	89
	U.S.A.	0	NA*	4^(^[18, 19, 26, 39]^)^	96
Study Quality (Newcastle-Ottawa Scale score)	High (5–6)	4^(^[11, 25, 36, 38]^)^	95	5^(^[18, 27, 36, 38, 39]^)^	90
	Moderate (3–4)	4^(^[8, 10, 17, 35]^)^	72	4^(^[12, 19, 26, 40]^)^	88

*NA Not applicable

Subgroup analysis of the GA and IM groups based on average age and proportion of males in the study population, and study quality criteria is displayed in Table 3. Of those studies that presented the data, four of seven studies of GA had a majority of female patients (incidence rate range 0.5 to 2.4 per 1000 person-years), however six of eight studies of IM had predominantly male patients (incidence rate range 0.38 to 17.1). Four of seven studies of GA had patients greater than 55 years of age, with a range of incidence rates 1.0 to 15.2 per 1000 person-years, with the three other studies presenting incidence rates between 0.5 and 2.4 per 1000 person-years. Seven of the nine IM studies had patients with an average age greater than 55, with a rate of incidence rates 1.3 to 17.1 per 1000 person-years, with the remaining two studies’ rates 0.38 and 4.1 per 1000 person-years. Using the abridged Newcastle-Ottawa Scale, of the GA publications there were four of high quality (incidence rate range: 0.53 to 15.24 per 1000 person-years), four of moderate quality (incidence rate range: 2.33 to 5.16 per 1000 person-years) and none that were of low quality. Of IM publications there were five high quality studies (incidence rate range: 0.38 to 4.10 per 1000 person-years), four moderate quality studies (incidence rate range: 2.16 to 17.08 per 1000 person-years), and no low quality studies.

### Publication bias

The funnel plots did not appear to show any obvious lack of symmetry and therefore were not indicative of publication bias, for the studies of both GA and IM (Additional file 2: Figure S1).

## Discussion

To our knowledge, this is the first systematic review to describe risk of patients with GA or IM developing GC, with these premalignant lesions diagnosed on histological examination. Our results show there is a wide variation in incidence rates in previous studies, including within continents. Also, due to poor study quality there is substantial heterogeneity.

There has been one previously published systematic review of the risk of GC in patients with GA, however diagnosis of GA was by serological methods [13]. We only included GA and IM lesions diagnosed via histology in the current review as, although serological methods using pepsinogen ratios is a recognised technique for diagnosis, histological evaluation remains the most commonly used method. Laboratories use different pepsinogen ratio cut-offs to diagnose GA which, introduces inconsistency and potentially misdiagnosis of GA lesions when compared to histological analysis, so although serological diagnosis is a useful method, it is subject to limitations [21].

Development of GA and IM commences a cascade of mucosal changes which can progress to GC, as described by Correa et al [22]. In some Asian countries, patients who are diagnosed with GA or IM enter into a surveillance programme and prescribed *H. pylori* eradication therapy, if positive on tissue biopsy [23]. However, in most Western countries there are no surveillance programmes and patients are often not followed up. Recent European guidelines now recommend patients with GA or IM affecting both the antrum and the corpus should undergo endoscopic follow-up, but not in those with lesions limited to the antrum [24]. As pepsinogen ratios cannot distinguish the extent of mucosal changes, histological diagnosis is required to determine patient qualification for entrance to this surveillance programme.

There was a large degree of heterogeneity between studies with GC, incidence rates ranging from 0.38 to 17.08 per 1000 person-years in individual studies, and high *I* ^2^ values when study estimates were pooled. Potential contributing factors include differences in study design, sample size and methods used to identify GC patients, which, when the Newcastle-Ottawa criteria are applied, result in a lower pooled GC incidence rate for studies of higher quality compared to analysis containing all studies, however high heterogeneity remains. Sensitivity analysis removing individual studies did not reduce this heterogeneity.

Causes for the high heterogeneity roots from study quality and consistency. Six of the 8 GA studies had incidence rates of between 1.0 and 5.2 per 1000 person-years, with the remaining two studies having more extreme results (0.53 and 15.24). There was a lack of detail regarding methods used to recruit patients for endoscopy, with only three studies reporting their techniques. Furthermore, there was a substantial variation in the amount of person-years follow up, ranging from 232 (which detected no gastric cancer cases) to 115,583. Of note, the studies producing the more extreme results had significantly less follow up than other studies (mean 33,076), which may have contributed to the wide range in incidence rates results. Takata et al. showed a much higher incidence rate than other studies (15.2 per 1000 person-years), a cause for which may include the outcome source [25]. This article does not describe how they collected data for the study and thus their techniques may be inconsistent with the others included in this review.

Similar to the studies of GA, two of the nine studies in the IM cohorts had extreme incidence rates (0.38 and 17.08 per 1000 person-years), [26, 27] whereas the remaining incidence rates were between 1.26 and 4.10 per 1000 person-years. The study by Kim et al. had a markedly different demographic than others whereby the average age of patients was 45 and there was a male predominance of 88%, in contrast to other studies where there was a more even distribution of age and gender [27]. Horsley-Silva’s study had a shorter follow up than seven of the eight other IM studies (820 person-years), contrasting with the mean (16,774 person years) of these remaining IM publications, potentially contributing to the high incidence rate [26].

Despite the significant increase in 5-year survival from GC in Japan, attributed to introduction of GC surveillance methods, a 2009 report indicated that introduction of such a programme was not feasible in the UK, [28]. This is at variance with recently published European guidelines which advise follow-up endoscopy, for extensive lesions [24]. Our study has shown that, compared with background risk of GC, patients with GA or IM in European countries are at greater risk of this tumour. These incidence rates are comparable with recent studies of oesophageal adenocarcinoma risk in patients with the premalignant lesion Barrett’s oesophagus, for whom there is currently an endoscopic surveillance programme [29, 30].

Consistent with published literature, we found study location impacted the rate of transformation to GC [31]. Patients with GA had a higher risk of GC in Asia, when compared to Europe, whereas, conversely, the IM group showed an increased incidence rate in European countries compared to the Asian continent. A lower rate of GC in patients with IM in Asia may reflect the introduction of surveillance programmes, however this was not found in patients with GA. As there was only one Asian study in the IM group, [27] further studies are required to confirm this lower rate. Significant variation in demographics and methodology in this study may contribute to the findings, such as the lower mean age of patients (45 years old).

*H. pylori* is the most common risk factor for GC development, often resulting in atrophy of the gastric mucosa, commencing a cascade toward carcinoma [32]. We were unable to perform a sensitivity analysis limited to studies that adjusted for *H. pylori* infection as those that included this risk factor in analysis described its prevalence but not adjustment in GC incidence calculation. Therefore, due to the prominent role *H. pylori* bacteria has in carcinogenesis, [33] further studies should include consideration of adjustment for *H. pylori* infection when determining GC incidence in patients with GA or IM.

A recognised limitation of systematic reviews includes the impact of demographic and within/between study methodology variation [34]. Furthermore, bias can arise from non-publication of smaller studies with non-statistically significant results. However, the funnel plot demonstrates that there is little evidence of such publication bias in this review. In addition, comparing the incidence rates of patients with GA or IM to the general population can be affected by study timing, since worldwide incidence rates of GC are decreasing; studies from 1974 (Siurala et al.) [35] may be less applicable compared to more recent publications. In addition, as only one publication reflecting two studies was population-based, [36] incidence rates from individual tertiary referral centres may not be representative of the entire population. A further weakness occurred in five studies where the total number of person-years was not described, but was estimated from the median number of person-years. Furthermore, there were several studies that determined GC in a GA/IM cohort but did not report sufficient information for the incidence rate to be calculated. Therefore, we recommend that future studies should report the total number of person years and GC incidence to enable comprehensive meta-analyses to be conducted. In addition, there were multiple studies that did not exclude GCs detected in the first 6 months post-initial endoscopy. This is an important factor in cancer diagnosis as a GC may have been present in the first endoscopy but not detected. Thus excluding GC diagnosed within the first 6 months post-endoscopy resulted in the number of GC cases being lower than described. It is known the level of heterogeneity is associated with the predictive value of meta-analyses, [37] and, thus due to the high heterogeneity in this study (>90%), when estimates are pooled an overall incidence rate is not presented. Significant heterogeneity affects reliability of combining results and thus we recommend future studies of this important subject is required in order to produce a reliable pooled incidence rate of cancer risk.

## Conclusion

In conclusion, this systematic review has shown the incidence rate of progression from GA and IM to GC varies by study geographical location. We also highlight the substantial heterogeneity between studies and that more robust studies are required so that reliable pooled estimates can be calculated. As this disease has a poor prognosis and is a common cause of death from cancer, influenced by its advanced stage at diagnosis, further research in this area is of importance.

## Additional files

Additional file 1: Table S1. Terms and database search strategy used in MEDLINE for systematic review. (DOCX 41 kb)

Additional file 2: Figure S1. Funnel plots to show potential publication bias for GA (left) and IM (right). (PNG 100 kb)

## Abbreviations

CI: Confidence interval
GA: Gastric atrophy
H.pylori: Helicobacter pylori
IM: Intestinal metaplasia
OC: Oesophageal cancer
