# Supplementary material for: Adenocarcinoma risk in gastric atrophy and intestinal metaplasia: a systematic review
Source: BMC Gastroenterol. 2017 Dec 11;17:157. doi: 10.1186/s12876-017-0708-4 (PMC5725642; doi:10.1186/s12876-017-0708-4)
Supplement: Supplementary file 1 — Terms and database search strategy used in MEDLINE for systematic review. (DOCX 41 kb) [file 12876_2017_708_MOESM1_ESM.docx]

Additional file 1 Table S1: Terms and database search strategy used in MEDLINE for systematic review

| Number | Terms |
| --- | --- |
| 1 | (gastric* OR stomach* or ‘?esophagogastric junction’ OR ‘cardio?esophageal junction*’) AND (cancer* OR tumo?r* OR neoplasm* OR carcinoma* OR adenocarcinoma*) |
| 2 | Stomach neoplasms |
| 3 | 1 OR 2 |
| 4 | Atrophy |
| 5 | Gastritis, atrophic |
| 6 | (intestin* AND metaplas*) |
| 7 | Helicobacter pylori |
| 8 | ‘h. pylori*’ |
| 9 | Pepsinogen* |
| 10 | 4 OR 5 OR 6 OR 7 OR 8 OR 9 |
| 11 | 3 AND 10 |
| 12 | Animals NOT (humans AND animals) |
| 13 | 11 NOT 12 |
| 14 | Limit 13 to ‘review’ |
| 15 | 13 NOT 14 |
