# Supplementary figures and images for: Adenocarcinoma risk in gastric atrophy and intestinal metaplasia: a systematic review
Source: BMC Gastroenterol. 2017 Dec 11;17:157. doi: 10.1186/s12876-017-0708-4 (PMC5725642; doi:10.1186/s12876-017-0708-4)

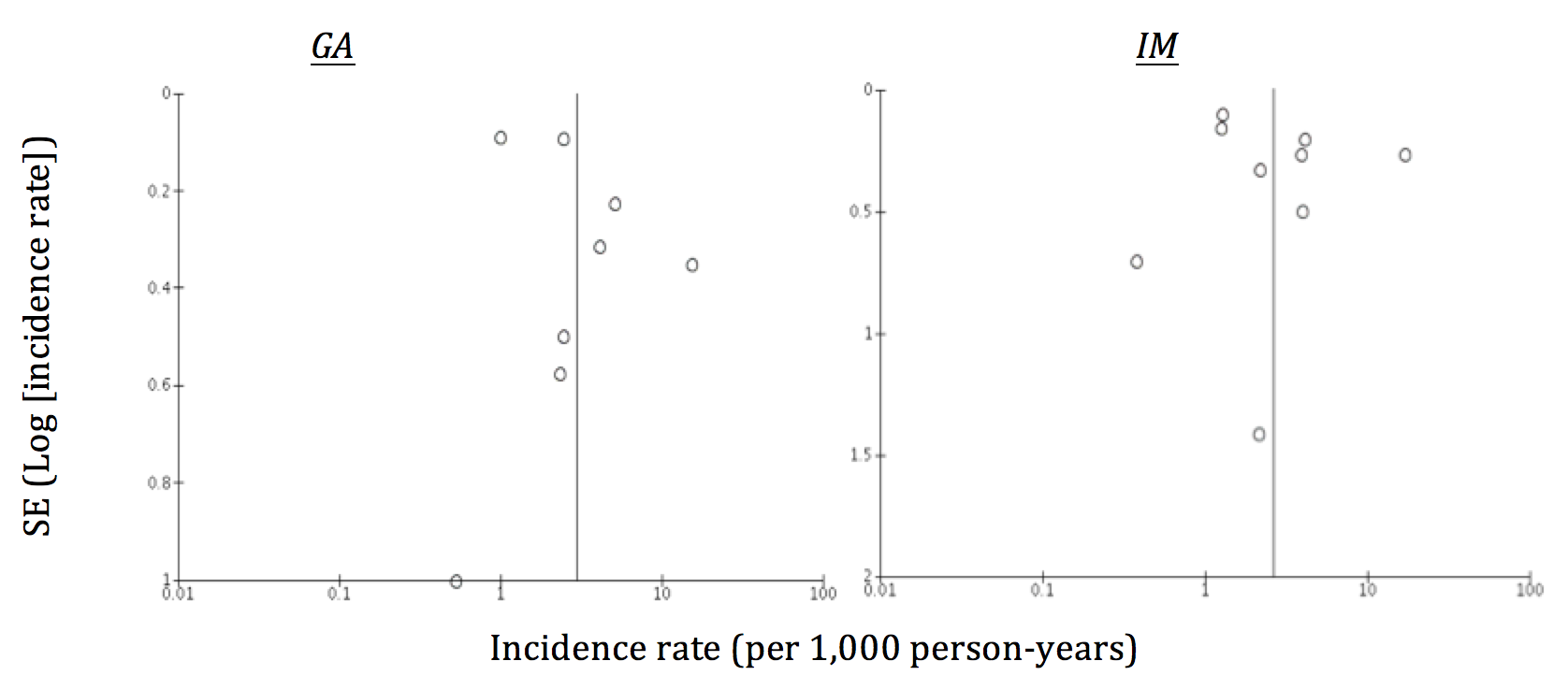

Supplement: Supplementary file 2 — Funnel plots to show potential publication bias for GA (left) and IM (right). (PNG 100 kb) [file 12876_2017_708_MOESM2_ESM.png]
